# Towards computational improvement of DNA database indexing and short DNA query searching

**DOI:** 10.1080/13102818.2014.959711

**Published:** 2014-10-31

**Authors:** Done Stojanov, Sašo Koceski, Aleksandra Mileva, Nataša Koceska, Cveta Martinovska Bande

**Affiliations:** ^a^ Faculty of Computer Science, Department of Computer Technologies and Intelligent Systems, University “Goce Delčev”, Štip, Republic of Macedonia

**Keywords:** DNA database, fast indexing and search, all hits, *E. coli*

## Abstract

In order to facilitate and speed up the search of massive DNA databases, the database is indexed at the beginning, employing a mapping function. By searching through the indexed data structure, exact query hits can be identified. If the database is searched against an annotated DNA query, such as a known promoter consensus sequence, then the starting locations and the number of potential genes can be determined. This is particularly relevant if unannotated DNA sequences have to be functionally annotated. However, indexing a massive DNA database and searching an indexed data structure with millions of entries is a time-demanding process. In this paper, we propose a fast DNA database indexing and searching approach, identifying all query hits in the database, without having to examine all entries in the indexed data structure, limiting the maximum length of a query that can be searched against the database. By applying the proposed indexing equation, the whole human genome could be indexed in 10 hours on a personal computer, under the assumption that there is enough RAM to store the indexed data structure. Analysing the methodology proposed by Reneker, we observed that hits at starting positions 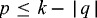 are not reported, if the database is searched against a query shorter than 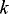 nucleotides, such that 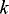 is the length of the DNA database words being mapped and 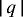 is the length of the query. A solution of this drawback is also presented.

## Introduction

Increased knowledge about the complex human genome is revealing its importance and impact on people's lives. The advances in computer science have contributed to the storage, dissemination, search and analysis of human genome data with increased efficiency and accuracy. That is why genetic databases are gaining more and more popularity in the research community today. Genetic databases often include entire genomes and could be used for searching particular sequences in genetic disease analysis, DNA fingerprinting, genetic genealogy or analysis of short sequences, such as non-standard codon structure [1] and codon context frequency.[2]

As soon as the first genetic database became available on the Internet, the necessity of fast DNA database-processing algorithms became a challenge that is still a challenge to researchers today. Being database inapplicable, dynamic programming-based solutions for global/local sequence alignment, such as Needleman–Wunsch [3] and Smith–Waterman,[4] have been substituted with faster, heuristic seed-based algorithms such as FASTA (Fast Alignment) [5] and BLAST (Basic Local Alignment Search Tool),[6] performed in two phases. In the first, so-called preprocessing phase, the matching positions of highly similar regions are identified as seeds, and in the second phase, the seeds are extended to local alignment. Usually, not every initial seed is extended to full alignment; instead, many of them are discarded by filtering, which results in lower run-time.

One common task – searching a genetic database to find exact matches for a non-degenerate or partially degenerate query – is usually done by using web applications hosted and run on remote web servers.[7] For smaller databases, computer desktop programs can be also used, with all the data kept in the main memory. The main feature of all these algorithms and tools is the phase of database indexing, which precedes and speeds up the actual searching phase. There are also algorithms for searching large genetic databases rapidly on desktop computers with limited RAM, like MICA (K-Mer Indexing with Compact Arrays),[8] which stores indexed data on a disk and retrieves relevant data selectively during the searching phase. Indexing a DNA sequence with MICA is achieved by dividing the sequence in chunks of (2^16^–1) bases, scanning each chunk with a window of width *K* and storing the positions of all overlapping *K*-mers in array.

One group of algorithms uses suffix trees (OASIS [9] and the three versions of MUMmer [10–12]), and enhanced suffix arrays (ESAs) (Vmatch [13]) for building database indexes. ESAs consist of four arrays (suffix, longest common prefix, child and suffix link arrays) that together reach the full expressiveness of suffix trees. Suffix trees and ESAs are time efficient with complexity of O(*n*) and space complexity of O(*n* log(*n*)). The Kurtz [14] implementation of suffix tree requires 17.25 bytes per base, which is translated to 50 GB for the human genome, while ESAs require 4–8 bytes per base. There are also sparse suffix arrays (SSA), which index every *K-*th (sparseness factor) suffix of the sequence.[15] Their sparseMEM tool is able to find maximal exact matches faster than the previous methods, while using less memory. The tool essaMEM [16] optimizes the previous method by supplementing SSAs with a sparse child array for large sparseness factors, and this is known as enhanced sparse suffix array (ESSA).

Other new strategies deploy compressed indexing techniques that reduce the space complexity to O(*n*) bits. Techniques include Ferragina–Manzini index (FM-index),[17] compressed suffix arrays (CSA) index [18] and Burrows–Wheeler transform (BWT) index.[19] For DNA sequences, BWT indexing was found to be the most efficient, and the memory requirement is less than 0.3 bytes per base. For the human genome, this requires only 1 GB memory and the whole index can reside in the main memory of a personal computer (PC). CSA index implemented with the BWT is used in [20,21] while BWT index is used in BWT-SW.[22] Another tool, backwardMEM,[23] uses enhanced CSA by indexing each *K*th suffix array value.

Benson [24] has proposed an algorithm for identification of all tandem repeats, without having to specify the pattern. Neglecting patterns that occur within a few database sequences, TEIRESIAS [25] is able to generate all maximal patterns that appear within at least 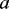 (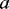 is user-defined) sequences. RAPID [26] is a probabilistic word-searching approach. A different significance is assigned to each match of length *k*, depending of the number of occurrences of the match. By partitioning the query and the subject sequence in fragments of fixed size, referred as windows, sequence search tree (SST) [27] can identify approximate matches, in time proportional to the logarithm of the database size.

Hash-based indexing strategies (SSAHA (Sequence Search and Alignment by Hashing Algorithm) [28,29] and BLAT (BLAST-like alignment tool) [30]), which are currently in more widespread use for DNA databases, require 1 byte or less per base, and they can be orders of magnitude faster than FASTA or BLAST, which index the query sequence rather than the database. SSAHA [28] partitions subject database sequence into non-overlapping words of length *k* (*k*-mers), being mapped into numbers according to the SSAHA conversion function. BLAT [30] builds up an index of non-overlapping *k*-mers and their positions in the database, excluding *k*-mers that occur too often from the index as well as *k*-mers containing ambiguity codes. During the search stage, three different strategies are used in BLAT, in order to find homologous regions: searching for perfect hits, allowing at least one mismatch between two hits and searching for multiple perfect matches which are in close proximity to each other. One problem with SSAHA and BLAT is their limitation, which rises from sacrificing the completeness for speed. They cannot detect matches with less than *k* bases.

Some conceptual and computational drawbacks of SSAHA have been solved by Reneker and Shyu.[29] According to Reneker and Shyu,[29] overlapping matches can be identified, if instead of indexing non-overlapping words of *k* bases, overlapping words of the same size are tracked in the indexed data structure. Reneker and Shyu [29] also pointed out that matches which are shorter than *k* bases can be identified as suffixes within some of the indexed words of *k* bases.

However, the previous concept is incomplete if some of the matches are located at the beginnings of the genetic sequences. In order to detect all matches, even the ones which are not reported in [29], we propose an improved searching methodology by integrating suffix search and prefix search that provide more exact matching.

From a computational viewpoint, we propose a computational upgrade of the indexing formula used by SSAHA and Reneker and Shyu,[29] which results in a *k*-fold speed-up of the indexing phase. The storage aspects were also improved due to the exclusion of redundant records from the indexed data structure. Instead of a hash table, a sorted dictionary indexed data structure is employed, which allows identification of all matches without having to scan all records, and hence, the better search time performance.

## Materials and methods

### Database indexing

Hash-based solutions, such as SSAHA and Reneker's improvements of SSAHA, are performed in two phases. In the first phase, DNA database words of *k* consecutive nucleotides 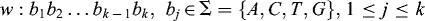, are mapped into numbers, applying a concrete base-mapping function. SSAHA and Reneker employ different base-mapping functions due to the fact that none of the nucleotides can be zero-mapped. According to SSAHA, adenine is zero-mapped, 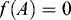. Since database words 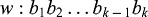 are mapped in integers as 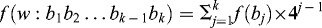, words ending with different number of **A**'s could not be distinguished if SSAHA's base-mapping function is used. For instance, since the mapped value of the words CAA and CAAAA is equal, 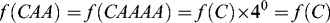, and they are assumed to be equal, which is incorrect. In order to overcome SSAHA's mapping inconsistency, Reneker and Shyu [29] proposed a modified base-mapping function: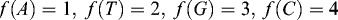 for distinction of words ending with different number of **A**'s.

The modified base-mapping function guarantees that a different number 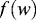 will be assigned for a different word, but neither SSAHA nor Reneker and Shyu's algorithm has been improved in terms of the time complexity of the indexing phase. Indexing complete genomes or recomputing the indexed data structure, if the DNA data has been modified, is a time-demanding process that could last even a few days, if it is executed on a PC. In these cases, there is a computational necessity to reduce the time span of the indexing phase. Therefore, we propose a computational upgrade of the indexing formula used in SSAHA and Reneker and Shyu's algorithm that speeds up the indexing *k*-fold, such that *k* is the length of the DNA words being mapped, in comparison to SSAHA and Reneker and Shyu's algorithm.

The proposed computational upgrade is based on the following. Once the first word from the *i*th DNA database sequence 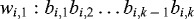 has been mapped, the mapped value of each successive word 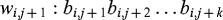 can be calculated from the previous one 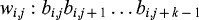, according to Equation (1). The equation is based on the fact that two successive words 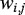 and 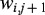 have exactly 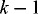 common nucleotides. Thus, each word 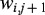 can be derived from the previous one 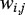, by excluding the leftmost nucleotide 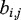
(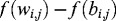 in Equation (1)), and by shifting the common nucleotides for one position to the left (division by 4) and addition of a new base 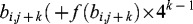 in Equation (1)):
(1) 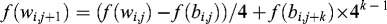



For instance, if AACTT… is a DNA sequence and *k* = 4, once the first word of four nucleotides AACT has been mapped, 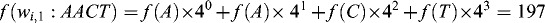, the mapped value of the following word *ACTT* can be calculated from the previous by applying Equation (1), 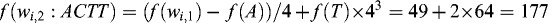 and so on.

Applying the mapping formula used in SSAHA and Reneker and Shyu's algorithm, 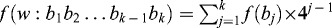, 4*k* operations are performed in order to map a single word. To map a DNA sequence 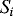 of *n* nucleotides, *n* − *k* + 1 words have to be mapped, i.e. 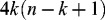 operations are performed. Since the length of the DNA sequence *n* is greater than *k* (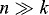), approximately 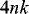 operations are performed in order to map all overlapping words of *k* nucleotides in 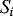.

Applying the proposed solution based on Equation (1), 4*k* operations are performed to map the first word only. Since the execution of Equation (1) requires four operations: subtraction (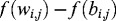), division (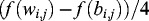), multiplication (
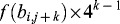
) and an addition (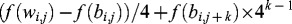) per mapped successive word, and there are 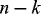 successive and overlapping words of *k* nucleotides in 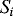, a total of 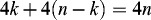 operations would have to be performed, which results in a *k*-fold increase in the speed of the indexing phase in comparison to SSAHA and Reneker and Shyu's approach.

SSAHA and the technique of Reneker and Shyu store indexed data differently. SSAHA precomputes a hash table of 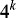 keys, such that 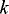 is the length of the DNA words being mapped. By generating 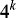 keys, SSAHA guarantees a different key for each different DNA word. Since the hash table is stored in the main memory, which has limited capacity, the application of SSAHA when running on a PC is suitable for indexing small databases. However, due to the fast processor–RAM communication, the time aspects of the indexing and search phase are relatively satisfactory.

Opposite to SSAHA, the indexed data can be stored in a file, which is kept on the disk or server, with much more storage capacity than the main memory. This idea, which is suitable for indexing long DNA sequences such as the human genome, has been employed by Reneker and Shyu.[29] However, the additional transfer of data between the RAM and the disk, during the indexing and searching phase, will slow down the time performance.

The idea of precomputing a hash table with 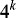 keys before tracking tuples 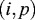, such that 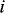 is the index of the DNA sequence where the word comes from and 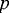 is its starting position, can be improved in terms of memory complexity. If a greater value is taken for 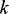, the number of precomputed keys in the hash table is increased. Since some of the precomputed keys may point zero 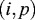 tuples, part of the main memory may be unnecessarily reserved.

In order to avoid situations in which memory is wasted for keys pointing zero 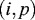 tuples, we propose the indexed data structure to be constructed dynamically. By reading and mapping overlapping words of 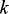 nucleotides found in the database, one can be sure that each key in the indexed data structure will point at least one tuple 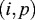.

For instance, if 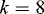 and the DNA word **TTTTCATT** is not contained in the database, then the key 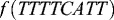 would be unnecessarily generated and kept in the memory. On the contrary, if the indexed data structure is constructed dynamically, by reading and mapping only words which are found in the database, a corresponding record key 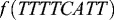 would not exist. This would contribute towards an optimization of the memory requirements.

If a short DNA database is indexed, by taking a large value for *k*, a significant part of the main memory would be wasted for keeping redundant keys corresponding to words which were not found in the database. This conceptual drawback of SSAHA not only implies unnecessary storage cost, but also slows down the search of a DNA pattern, since redundant keys are also examined in the searching phase. By reducing the size of the indexed data structure, better search time performance is expected, which may be of particular importance if an indexed data structure that tracks genomic data has to be searched.

### DNA pattern search

A database of DNA sequences is primarily searched as part of molecular biology studies, with the main purpose of finding genes, identification of regulatory sequences, predicting intron–exon structures of genes, analysis of short tandem repeats, pseudogenes recognition, finding restriction sites, etc. In some of the cases, such as prediction of a promoter sequence based on a known consensus element, biological information can be attached to the sequences and the database can be searched against relatively short DNA patterns.

In order to speed up the search of the indexed DNA database stored in the main memory, instead of a hash table, we use a sorted dictionary *SD* data structure. Due to the fact that records are sorted in ascending order of the keys, all hits can be identified without having to scan the entire indexed data structure, which is going to improve the search time complexity.

Given the set of short DNA patterns 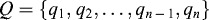 searched against the database, database words of 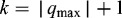 nucleotides, such that 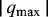 is the length of the longest query in 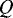, are mapped in keys pointing tuples 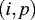, such that 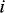 is the index of the database sequence from where the word has been read and 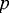 is the starting position of the word in the sequence. In such constellations, all query hits can be found either as suffixes or as prefixes within key-mapped words of 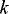 nucleotides. Also, note that the sorted dictionary is constructed by applying the proposed computational upgrade of the indexing formula used in SSAHA and the method of Reneker and Shyu.

Pattern 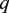 hit at starting position 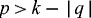 in the *i*th DNA sequence, such that 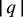 is the length of the pattern, is found as a suffix of a key-mapped word from the same sequence. For illustration, the query 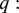 **AA** is found as a suffix in **TTAA**, being the second key-mapped word from the DNA sequence S_2_:C**TTAA**C…, if *k* = 4. The exact starting position of the hit can be derived from the tuple 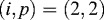, pointed by the sorted dictionary key 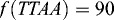, by increasing *p* for 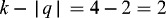, i.e. the tuple 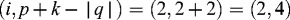 is reported.

An extension of the DNA pattern 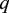 up to length 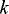 with a minimum value of conversion 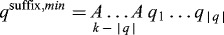 is obtained by adding 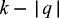 **A**'s to the left side. By adding 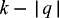 **C**'s also to the left side of the pattern, an extension of the same length, but with maximum value of conversion 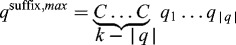 is obtained.

Each sorted dictionary ***key***, such that Equation (2) is satisfied, points tuple (tuples)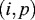 tracking words that contain the searched DNA pattern as a suffix. Hits are reported as 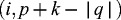 tuples, such that 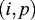 is a tuple pointed by a ***key*** that satisfies the following equation:
(2) 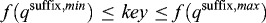



Each sorted dictionary ***key***, such that 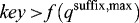, does not map a word that ends up with the searched DNA pattern. Based on this, all hits at starting positions 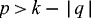 can be found, until the first ***key***, such that 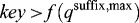 is read. None of the sorted dictionary records, such that 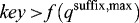, have to be considered. Thus, better search time performance is expected compared to SSAHA.

The main computational drawback of the improvements of SSAHA proposed by Reneker and Shyu [29] is the inability to find DNA patterns that are located at the very beginnings of the DNA sequences which were indexed. Namely, DNA pattern hits at starting positions 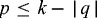, such that 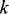 is the length of the words being mapped and 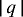 is the length of the searched DNA pattern, are not reported by the algorithm of Reneker and Shyu. This drawback might result in an incomplete identification of repetitive nucleotide sequences within telomeres if chromosomes are searched in a reverse direction, and in an inability to detect a key DNA pattern, such as promoter consensus, within short DNA reads.

Therefore, we propose a solution of the previous drawback, based on checking whether the sorted dictionary ***key*** satisfies Equations (3) and (4). If the value of the ***key*** is in the range between 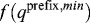 and 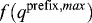, such that 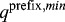 and 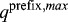 are obtained by addition of 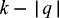 **A**'s and **C**'s to the right side of the searched DNA pattern (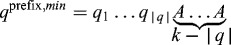, 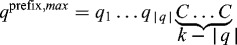) and the remainder when dividing 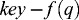 by 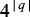 equals zero, then tuple (tuples) 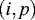 pointed by the ***key***, track mapped words that contain the searched DNA pattern as a prefix. Reported tuples 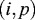 identify DNA pattern hits unreported by the algorithm of Reneker and Shyu:
(3) 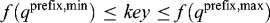

(4) 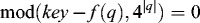



**Figure 1.**
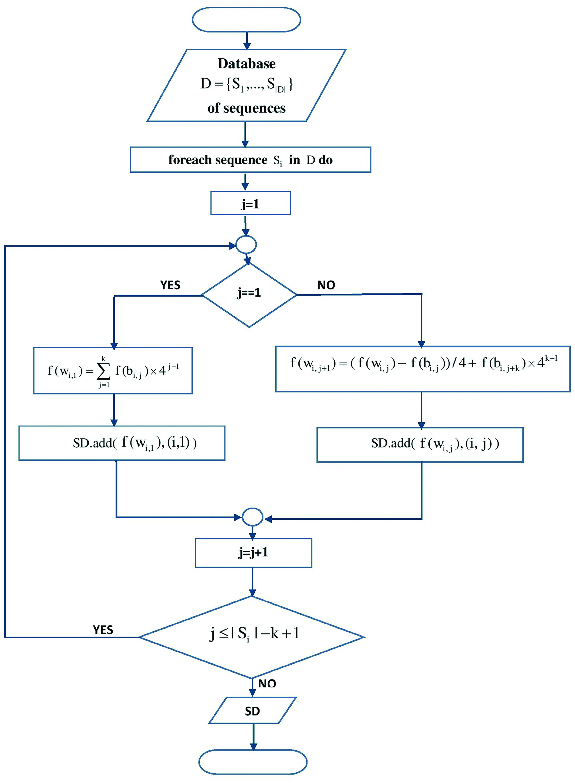
DNA data-indexing phase.

**Figure 2.**
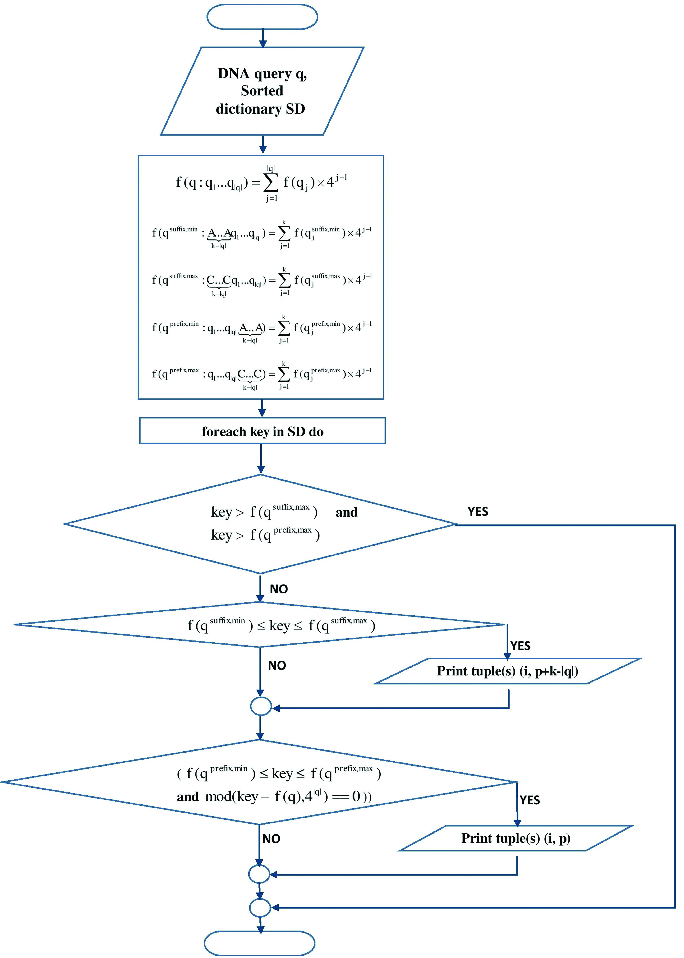
DNA pattern searching phase.

The flowcharts of the indexing and searching phase are shown in Figures 1 and 2.

### System characteristics

The proposed computational and conceptual upgrades were implemented in C# and tested on a Fujitsu Siemens computer with Core(TM) 2 Duo CPU at 2.67 GHz and 2 GB RAM.

### Database used

To evaluate the *computational*, *storage* and *matching* performances of the proposed approach, different *Escherichia coli* DNA fragments of a total size of 0.1 Gb (giga bases) retrieved from the European Nucleotide Archive,[31] were indexed and the result was compared to that obtained by SSAHA and Reneker and Shyu's algorithm, using the same computational resources.

## Results and discussion

**Figure 3.**
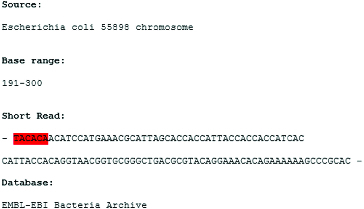
Short-read form *E. coli* 55989 chromosome, base range: 191–300.

Let us consider the short-read form *E. coli 55989 chromosome* in the base range 191–300 (Figure 3). If 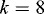, a search of the short read for *E. coli thrL* gene promoter consensus **TACACA**, which is located at the **−10** position relatively to the TSS (transcription start site), by the algorithm of Reneker and Shyu would not report the hit at the beginning of the short read. This is because when this algorithm is applied for a DNA pattern search shorter than *k* nucleotides, only hits that are found as suffixes of mapped words are reported. The consensus sequence **TACACA** is located at the start of the short read (Figure 3) and none of the mapped overlapping words of 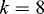 nucleotides: **TACACAAC**, **ACACAACA**, **CACAACAT…**, contain the searched DNA pattern **TACACA** as a suffix. Therefore, the **TACACA** hit at position 0 in the short read is not reported by Reneker and Shyu's approach.

The improvement proposed by us incorporates not only a suffix search strategy, but also a prefix search strategy based on Equations (3) and (4) and, thus, the hit at the beginning of the short read is reported. Before performing any check, 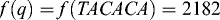, 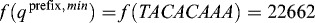 and 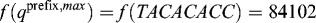 are computed. When the search comes to the sorted dictionary ***key*** = 71814, which corresponds to the mapped value of the word **TACACAAC**, and which contains the searched DNA pattern as a prefix, Equations (3) and (4) are satisfied: 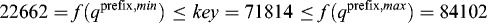 and 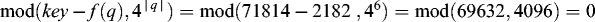. The consensus hit at the beginning of the short chromosome read is reported, represented with the tuple 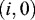, given that the short read is the *i*th DNA sequence being indexed.

By applying the proposed computational upgrade of the indexing formula used in SSAHA and Reneker and Shyu's algorithm, the complete *E. coli 55989* chromosome, which contains 5 Mb (mega bases), retrieved from the European Nucleotide Archive was indexed for one minute. Excluding the computational upgrade, eight minutes were spent for the same purpose, given that all *E. coli 55989* overlapping words of *k* = 8 nucleotides were mapped. Since the time complexity of the proposed computational upgrade is linear and the entire *E. coli* DNA data-set of 0.1 Gb was indexed in 20 minutes, the whole human genome, containing approximately 3 Gb, could be indexed in 600 minutes (10 hours). Using the same computational resources, the straightforward application of the indexing formula proposed by Reneker and Shyu would require 3.3 days. When compared to Simpson and Durbin,[32] who estimated that 4.5 days and 700 GB RAM would be required in order to index the human genome, the proposed computational upgrade results in an 11-fold speed-up.

The storage improvement was also experimentally analysed. For instance, given that the length of the mapped words equals 8, SSAHA pre-computes a hash table with 
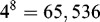
 keys. If the indexed data structure is constructed dynamically as proposed, the number of records in the data structure increases with the size of the indexed DNA data (Table 1). Using the same computational resources, the number of records in the indexed data structure was determined for indexing of 1, 2, 3, 4 and 5 Mb, extracted from *E. coli 55989* chromosome (Table 1). According to the results obtained, 64,422 records were tracked in the indexed data structure for 1 Mb DNA. The number of records in the indexed data structure increased to 65,471 records, which were tracked when the entire *E. coli 55989* chromosome was mapped. Even in that case, SSAHA unnecessarily keeps 65,536 − 65,471 = 65 keys, which map words of eight nucleotides that were not found in *E. coli 55989* chromosome. These 65 records are excluded from the indexed data structure, if the indexed data structure is constructed dynamically as we propose.

**Table 1.** Comparison of the number of records in the indexed data structures.

Base range (Mb)	SSAHA	Dynamic construction of the indexed data structure	Number of redundant records
1	65,536	64,422	1114
2	65,536	65,147	389
3	65,536	65,346	190
4	65,536	65,424	112
5	65,536	65,471	65

**Figure 4.**
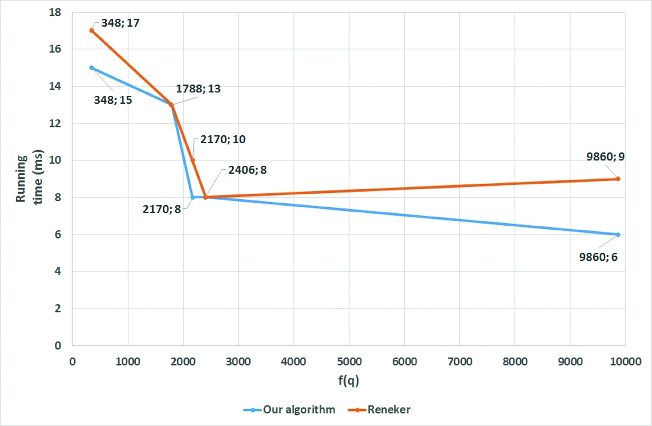
Comparison of the results in Table 2.

The use of a sorted dictionary instead of a hash table enables faster identification of all DNA pattern hits. In three out of the five cases, when searching for different promoter consensus sequences recognized by 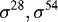 and 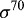 transcription factors (Table 2 and Figure 4), our algorithm ran faster than the one of Reneker and Shyu. The improvement is due to the computational concept of the proposed searching approach, which is able to identify the same DNA pattern hits as the one of Reneker and Shyu, but without having to examine all records in the indexed data structure. It is also noteworthy that the higher the conversion value of the searched DNA pattern is, the less records are examined, resulting in better search time performance.

**Table 2.** Comparison of the running times for searching different promoter consensus sequences

Sigma factor	Consensus query	Conversion value	Reneker and Shyu (ms)	Our algorithm (ms)
	CCGATAT	*f*(CCGATAT) = 9860	9	6
	TATAAT	*f*(TATAAT) = 2406	8	8
	TTGACA	*f*(TTGACA) = 2170	10	8
	CTGGTA	*f*(CTGGTA) = 1788	13	13
	CTAAA	f(CTAAA) = 348	17	15

In addition to the computational improvements, the main conceptual improvement of the proposed methodology, in comparison to the method of Reneker and Shyu, is the ability for detection of all DNA pattern hits, regardless of their starting positions in the sequences. There are no reports until now that SSAHA and the algorithm of Reneker and Shyu are not able to detect DNA pattern hits shorter than *k* nucleotides, which are located at the beginnings of the sequences. This drawback can be solved by employing a prefix search that provides more exact matching in comparison to Reneker and Shyu's algorithm (Table 3).

**Table 3.** Comparison of the matching rates

Sigma factor	Consensus query	Conversion value	Number of hits – Reneker and Shyu	Number of hits – our algorithm	Number of hits unreported by Reneker and Shyu
	CCGATAT	*f*(CCGATAT) = 9860	2520	2526	6
	TATAAT	*f*(TATAAT) = 2406	3812	3819	7
	TTGACA	*f*(TTGACA) = 2170	4010	4019	9
	CTGGTA	*f*(CTGGTA) = 1788	12,001	12,018	17
	CTAAA	*f*(CTAAA) = 348	18,140	18,166	26

According to the data in Table 3, the number of unidentified hits, when searching the indexed data structure for consensus elements **CCGATAT**, **TATAAT**, **TTGACA**, **CTGGTA** and **CTAAA**, ranges between 6 and 26. By applying the proposed methodology, a total of 65 unreported consensus hits located at the beginning of the indexed *E. coli* DNA fragments were additionally identified (Table 3).

Taken together, the results demonstrate that the improvements proposed by us give better *computational*, *storage* and *matching* performances in comparison to SSAHA and Reneker and Shyu's algorithm, using the same computational resources. To summarize the obtained results, Table 4 lists the advantages of the methodology proposed by us in comparison to the other two algorithms.

**Table 4.** Summary of the advantages of the presented methodology

Factor	SSAHA	Reneker and Shyu	Our algorithm
Time complexity of the indexing phase	O(*nk*)	O(*nk*)	O(*n*) – *k* times faster
Structure of indexed data structure	Hash table with 4*^k^* records	File	Sorted dictionary with less than 4*^k^* records for a small DNA database
Matching rate	Reports limited number of hits	Better matching rate than SSAHA, but unable to detect hits at the beginnings of the sequences	Identifies all DNA pattern hits

The biological impact of the proposed methodology lies in the ability to detect all DNA pattern hits, regardless of their starting positions in the sequences. Unlike SSAHA and the approach of Renker and Shyu, our algorithm can identify DNA pattern hits which are located at the beginnings of the indexed DNA sequences, by integrating suffix search and prefix search. As a consequence, the algorithm proposed by us is expected to provide more precise matching in DNA pattern analyses by being able to identify key DNA pattern hits that might indicate functional DNA data unreported by SSAHA and Reneker and Shyu's method.

## Conclusions

This work presents suggestions for computational and conceptual improvements of the most commonly used hash-based implementations for DNA database indexing and searching, such as SSAHA and Reneker and Shyu's improvements of the SSAHA software tool. We propose a computational upgrade of the indexing formula used in SSAHA and the algorithm of Reneker and Shyu, by which the database can be indexed *k*-times faster. This is of particular importance if large eukaryotic DNA sequences have to be tracked. Another improvement of our algorithm as compared to SSAHA is more efficient memory use when a relatively short DNA database is indexed: by dynamic construction of the indexed data structure, all redundant keys are excluded (i.e. those to which correspond hash values of words not found in the database), which improves the storage aspects. In addition, we use a sorted dictionary instead of a hash table as an indexed data structure, in order to be able to identify the same hits as Renker and Shyu's algorithm but without having to scan the entire data structure. As a result, the proposed methodology was demonstrated to run faster than Reneker and Shyu's algorithm. These better *computational*, *storage* and *matching* results as compared to SSAHA and Reneker and Shyu's approach, when using the same computational resources, indicate that our algorithm can be considered a promising improvement.
